# A rare case of t(11;22) in a mantle cell lymphoma like B-cell neoplasia resulting in a fusion of *IGL *and *CCND1*: case report

**DOI:** 10.1186/1755-8166-4-8

**Published:** 2011-04-01

**Authors:** Cristiano Krings Rocha, Inka Praulich, Iris Gehrke, Michael Hallek, Karl-Anton Kreuzer

**Affiliations:** 1 Department I of Internal Medicine, University at Cologne, Cologne, Germany

## Abstract

The chromosomal translocation (11;14)(q13;q32) rearranging the locus for cyclin D1 (*CCND1*) to that of the immunoglobulin heavy chain (*IGH*) can be found in virtually all cases of mantle cell lymphoma (MCL), while other *CCND1 *translocations are extremely rare. As *CCND1 *overexpression and activation is a hallmark of MCL it is regarded as a central biological mechanism in the development and maintenance of this disease.

Here we present a patient initially diagnosed with chronic lymphocytic leukemia (CLL) where chromosome banding analysis revealed, among other aberrations, a translocation (11;22)(q13;q11.2). We show by fluorescence in situ hybridization (FISH) analysis that on chromosome 22 the immunoglobulin light chain lambda (*IGL*) is involved in this cytogenetic aberration. Additionally, we demonstrate the resulting overexpression of *CCND1 *on the RNA and protein level, thereby consolidating the new diagnosis of a MCL-like B-cell neoplasia. Summing up, we described a rare case of t(11;22)(q13;q11.2) in a MCL-like neoplasia and showed that this aberration leads to an overexpression of *CCND1 *which is regarded as a key biological feature in MCL. This case underlines the importance of cytogenetic analyses especially in atypical cases of B cell lymphomas.

## Background

Mantle cell lymphoma (MCL) and chronic lymphocytic leukaemia (CLL) belong to the group of CD5-positive small B-cell neoplasms. While CLL is the most common leukaemia in western countries with an incidence rate of about 5 cases per 100,000, MCL is much less frequent with a 10-fold lower incidence rate of about 0.5 cases per 100,000 [1]. CLL and MCL share a distinct set of overlapping morphological features and clinical manifestation making it difficult in given situations to distinguish both diseases. Besides flow cytometric immunophenotyping, cytogenetic analysis is a deciding factor to discriminate between both entities. A prominent marker for MCL is the translocation (11;14)(q13;q32), resulting in a rearrangement of the gene loci for immunoglobulin heavy chain (*IGH*) on chromosome 14 and cyclin D1 (*CCND1*) on chromosome 11. This leads to a constitutional overexpresion of *CCND1 *due to the *IGH *enhancer sequence located in front of *CCND1 *[2]. Cyclin D1 is a cell cycle regulator usually transiently expressed in cells. Its aberrant overexpression in MCL leads to a high mitotic rate of the affected B-cells [3]. Other translocations resulting in an increased *CCND1 *gene expression are extremely rare [4-6].

In CLL cytogenetic studies focus on fluorescence in-situ-hybridization (FISH) analyses for trisomy 12, deletion of 6q, 11q, 13q and 17p, although about 30% of CLL cases show non-recurrent translocations [7]. The lack of mitotic CLL cells under culture conditions has largely been limiting the use of other chromosome analysis techniques. Recently, the use of new stimulating substances like CpG-oligonucleotide DSP-30 and Interleukin-2 resulted in an increase of proliferating CLL cells in culture and remarkably improved the success rate of classical chromosome analysis in CLL diagnostics [8-10]. Since then many cases of CLL where reported showing atypical and rare chromosomal aberrations, making the distinction borders to other B-cell malignancies blurry [11,12].

Despite these significant improvements, flow cytometric immunophenotyping remains the most important diagnostic tool for diagnosis of different B-cell neoplasms. While bright expression of FMC-7 and surface immunoglobulin (sIg) are usually seen in MCL but absent in CLL, the surface marker CD23 is typically found on CLL cells, but not MCL cells. Cases of CLL with variant phenotypes negative for CD23 and/or positive for FMC-7 usually are tested for t(11;14)(q13;q32) to eliminate the possibility of misdiagnosing MCL [13]. Since the clinical course of MCL is very aggressive with an overall survival of 3 to 5 years, all possible efforts for distinguishing CLL from MCL should be done.

## Case presentation

In March 2010 we received peripheral blood (PB) from a 60 year old woman initially diagnosed with CLL in the context of a clinical CLL trial. The patient was therapeutically naive and exhibited an initial lymphocyte count of 22 × 10^6^/ml. By cell morphology a blastoid MCL variant could be excluded. Clinically, a general lymphadenopathy but no B symptoms were present. So far the disease developed slowly over 24 months suggesting an intermediate progressive course. The IgVH status was mutated. Unfortunately, no bone marrow or lymph node biopsy was available for further analysis.

## Materials and methods

### Flow cytometry

Five-color flow cytometric analysis was performed on a FC500 instrument (Beckman Coulter, Brea/CA, USA) as described by Costa et al. [14]. The following antibodies were used: CD19-ECD, CD5-FITC, CD10-PE, CD23-FITC, FMC7-FITC, CD79b-PE (Beckman Coulter, Brea/CA, USA) and polyclonal rabbit anti-human lambda light chains, rabbit F(ab')2 PE-conjugated (DAKO, Glostrup, Denmark).

### Chromosome analysis

Peripheral blood was cultured for 96 hours in MarrowMax medium (Invitrogen, Grand Island/NY, USA). Two cultures with different mitogens were used: 12-*O*-tetradecanoylphorbol-19-acetate (Sigma-Aldrich, St. Louis, USA) and CpG-oligonucleotide DSP30 (TIB MOLBIOL, Berlin, Germany) plus Interleukin 2 (Biochrom, Berlin, Germany). Chromosome analysis was performed on trypsin giemsa banded chromosome preparations and karyotypes were interpreted according to the ISCN 2009 [15].

### Fluorescence in-situ-hybridization (FISH)

Interphase FISH was performed with a commercial set of probes consisting of 13q14/13q34 (D13S25, D13S319/13q34), 11q22/11cen (ATM/D11Z1), 6q23/6cen (MYB/D6Z1), 17p13/17cen (TP53/D17Z1) and centromere 12 (D12Z3) (MetaSystems, Altlussheim, Germany). Additionally, dual-color, dual-fusion translocation probes for CCND1/IGH and for BCR/ABL were used (MetaSystems, Altlussheim, Germany). The gene rearrangement involving *IGL *was assessed using an IGL dual-color, breakapart probe (MetaSystems, Altlussheim, Germany). The CCND1/IGL translocation was analysed with a tri-color, dual fusion probe consisting of a CCND1 dual color, break-apart probe (Abbott Molecular, Downers Grove, IL) and a homebrew IGL probe labeled in Spectrum Aqua (CTA-526G4, CTA-60B5, CTA-865E9). Interphase and metaphase FISH analysis was performed according to the manufacturer's instructions.

Multicolor FISH (mFISH) was performed on metaphase spreads using the 24XCyte MetaSystems Chromosome painting kit according to the manufacturer's instructions (MetaSystems, Altlussheim, Germany).

### Immunobloting

Cells were lysed with MPER Mammalian Extraction Reagent (Thermo Scientific, Rockford/IL, USA) including DTT on ice for 60 minutes. Protein concentration was measured by Bradford assay. Solubilised proteins were resolved by PAGE and transferred onto a nitrocellulose membrane (Invitrogen, Karlsruhe, Germany). Blots were probed with monoclonal mouse anti-human cyclin D1 antibody (BD Biosciences, Franklin Lakes/NJ, USA), monoclonal mouse anti-ß-actin antibody (Sigma-Aldrich, USA) and a polyclonal goat anti-mouse peroxidase-conjugated secondary antibody (Dako, Denmark). Antibody binding was detected using Amersham ECL™Western blotting detection reagents (GE Healthcare UK Limited, Buckinghamshire, UK). The well-characterized MCL cell line GRANTA-519, which features the t(11;14)(q13;q32), hence shows a high expression of cyclin D1 protein [16] served as a positive control.

### Quantitative real-time RT-PCR

For quantitative real-time RT-PCR, total RNA from the patient and GRANTA-519 cells was purified using QIAmp RNA Blood Mini Kit (Qiagen, Hilden, Germany) and cDNA synthesis was subsequently done applying First Strand cDNA Synthesis Kit (Roche, Grenzach-Wyhlen, Germany). The expression status of cyclin D1 (*CCND1*) was monitored using TaqMan© Probe real-time PCR assay with LightCycler© FastStart DNA MasterPLUS HybProbe Kit (Roche, Grenzach-Wyhlen, Germany) on a LightCycler© 480 Instrument. For amplification of *CCND1*, forward primer 5'-AGTGCAAGGCCTGAACCTG-3', reverse primer 5'-GGCAGTCTGGGTCACACTTGA-3' and the probe 5'-6FAM-TTCCTGTCCTACTACCGCCTCACACGCTTC-Dabcyl-3' were applied with a standard PCR cycling profile of 45 cycles and an annealing temperature of 60°C. Target gene expression was normalized against the expression of the housekeeping gene *ABL1 *with primers and a probe as previously described [17] and target gene expression values are given as *%CCND1*/*ABL1 *with PCR-efficiencies for *CCND1 *and *ABL1 *of 2,00 and 1,99, respectively.

## Results

**Figure 1:**
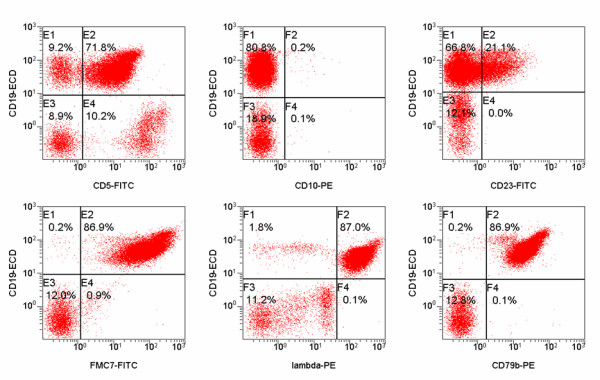
**Flow cytometry results**. Flow cytometric graphs showing positivity for CD5, CD79b, FMC7, surface immunoglobulin lambda and negativity for CD23 and CD10.

**Figure 2:**
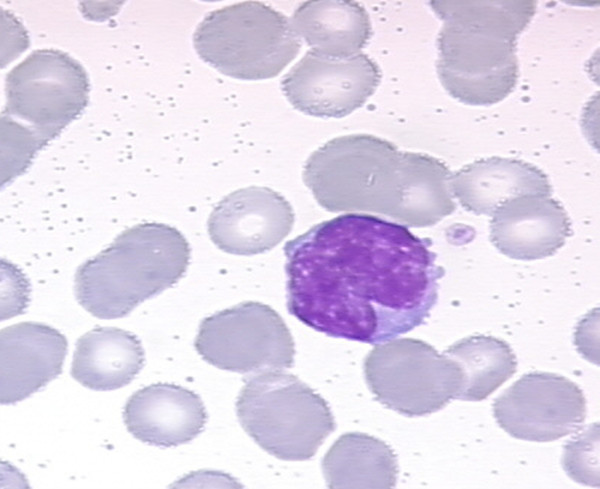
**Morphology of patient's leukaemic cells**. Medium-sized lymphoid cell with lightly basophilic cytoplasm and medium-sized irregular nucleus with dispersed chromatin.

**Figure 3:**
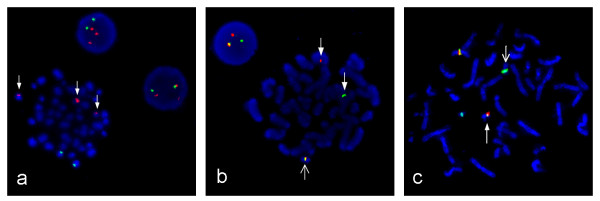
**Metaphase and interphase FISH**. **(a) **FISH showing 3 signals for *CCND1 *due to a translocation at the *CCND1 *locus on 11(q13). *CCND1 *on 11(q13) is marked in red, *IGH *on 14(q32) is marked in green. **(b) **FISH confirming *IGL *rearrangement with an *IGL *dual-color breakapart probe. Broad arrows show separated signals for the *IGL *proximal region in red and for the distal region in green. The open arrow shows a fusion signal for the normal chromosome 22. **(c) **FISH with a *CCND1/IGL *tri-color, dual-fusion probe, showing the *IGL/CCND1 *translocation. *CCND1 *on 11(q13) marked in Spectrum Orange and Spectrum Green, *IGL *on 22(q11.2) marked in Spectrum Aqua. The broad arrow shows the derivative chromosome 22 and the open arrow the derivative chromosome 11.

**Figure 4:**
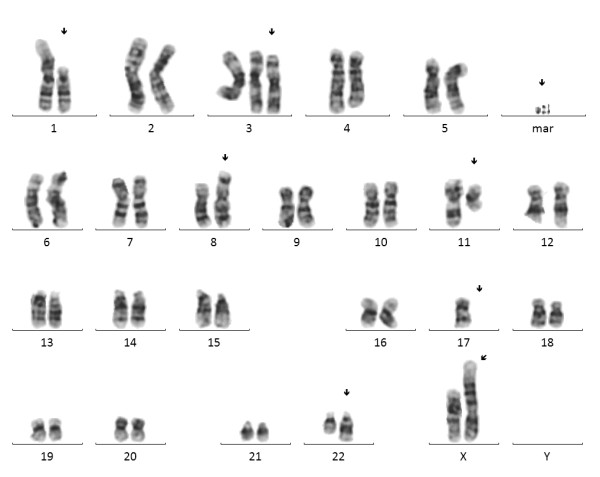
**Conventional chromosome analysis**. Complex aberrant karyogram of patient showing the karyotype 46, X, der(X)t(X;1)(p22.1;p21), del(1)(p21), +3, der(8)t(8;17)(p21;q21), t(11;22)(q13;q11.2), -17, 3dmin.

**Figure 5:**
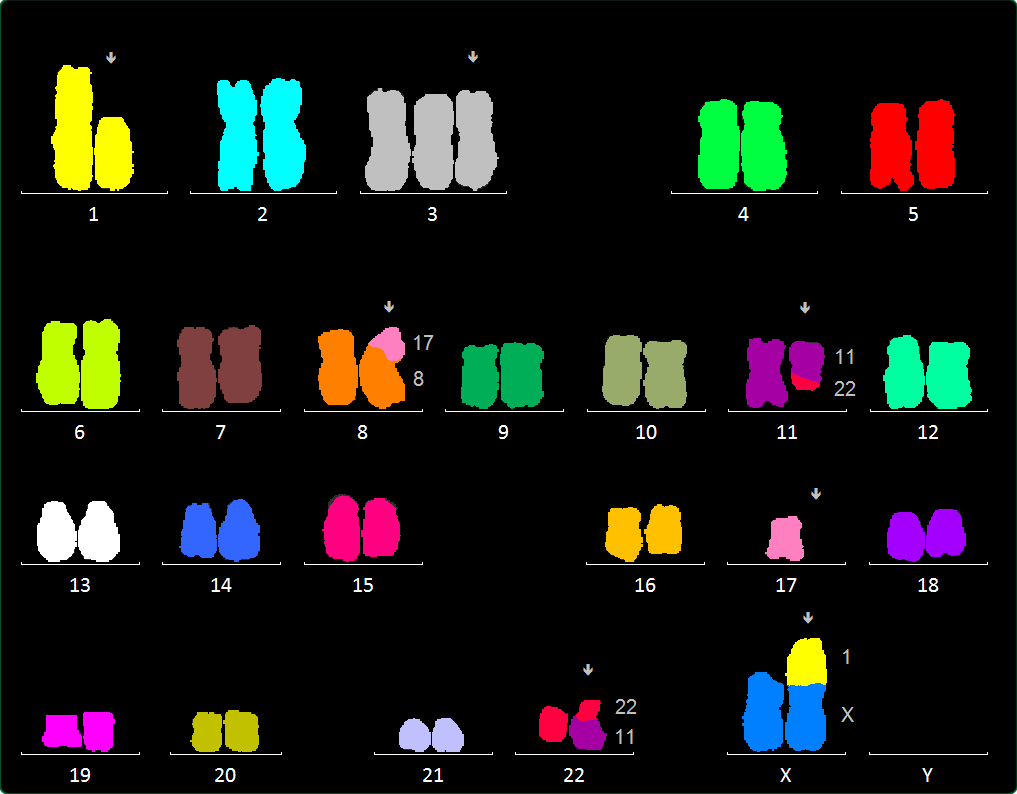
**mFISH analysis**. Multicolor FISH analysis confirmed the deletion on chromosome 1, the trisomy 3, the unbalanced translocation (8;17), the balanced translocation (11;22), the loss of one chromosome 17 and the unbalanced translocation (X;1).

Flow cytometric analysis showed a positive population for CD5, FMC-7, CD79b and surface Ig lambda but negativity for CD10 and low CD23 (Figure 1). This immunophenotype was more consistent with a MCL than with CLL. Morphology on peripheral blood smears showed no smudge or basket cells which are typical for CLL patients. Instead lymphoid cells with irregular nuclear contours were seen, fitting the antecedent suspicion of MCL (Figure 2). For further confirmation of the supposed MCL a FISH for t(11;14)(q13;q32) was performed. Surprisingly, the rearrangement of *IGH *and *CCND1 *could not be confirmed, but an additional signal for 11q13 was observed in 78% of analysed cells (Figure 3a). Two constellations were possible: Trisomy 11 or translocation of chromosome 11 involving the *CCND1 *gene. Chromosome analysis showed two different clones, both with a trisomy 3 and a translocation t(11;22)(q13;q11.2), proving the second constellation to be true. Karyotype was confirmed via mFISH and described as 47, XX, +3, t(11;22)(q13;q11.2) [16]/46, X, der(X)t(X;1)(p22.1;p21), del(1)(p21), +3, der(8)t(8;17)(p21;q21), t(11;22)(q13;q11.2),-17,2~9dmin [4] (Figure 4 and 5).

Further analysis applying our routinely used CLL FISH-panel showed a normal signal pattern for 6q23/6cen, 13q14/13q34, and centromere 12. Interphase FISH for 11q22/11cen had a normal signal pattern, whereas on metaphases the translocation t(11;22) could be confirmed, given that signal for 11q22 and for 11 centromere were localized on different chromosomes (data not shown). Interphase and metaphase FISH for 17p13/17cen were aberrant on cells with the complex clone showing one signal for 17p13 (aberrant in 10% of analysed interphases), well-fitting the unbalanced translocation t(8;17) with loss of 17p (data not shown).

To determine the translocation partner gene for *CCND1 *on chromosome 22 three different FISH probes were used: one dual-color, dual-fusion probe for *BCR/ABL*, a dual-color, breakapart *IGL *probe and a tri-color, dual fusion CCND1/IGL probe. The *BCR/ABL *probe showed a normal signal pattern (data not shown) whereas the *IGL *probe showed a break in one *IGL *gene locus in 84% of analysed cells (Figure 3b). FISH analysis with the *CCND1/IGL *tri-color, dual fusion probe could clearly show the CCND1/IGL translocation on 78% of analysed interphases and also on metaphases (Figure 3c).

**Figure 6:**
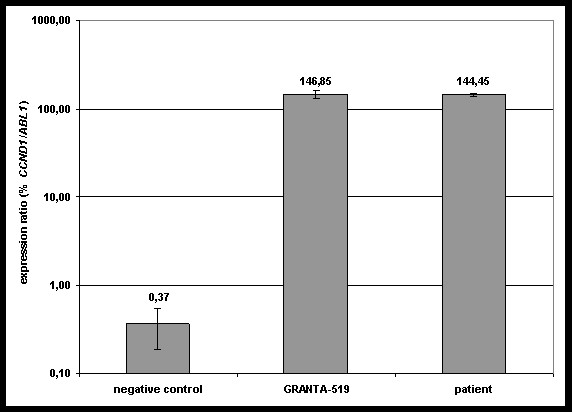
**Quantitative real-time RT-PCR results**. The overexpression of *CCND1 *could be clearly detected for the patient in comparison to GRANTA-519 *CCND1*-positive cells. Expression ratios are given as %*CCND1/ABL1 *in log-scale on the y axis.

**Figure 7:**
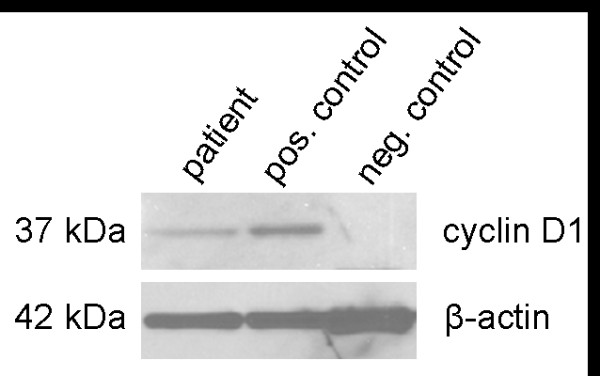
**Western blot analysis of CCND1 expression**. The overexpression of cyclin D1 in our patient is clearly visible (left panel) as well as in GRANTA-519 cells with t(11;14)(q13;q32) (middle panel) compared to a healthy donor (right panel).

As investigated by quantitative real-time RT-PCR, an overexpression of *CCND1 *could be clearly detected for the patient (*%CCND1*/*ABL1 *= 144,45) compared to GRANTA-519 cells which served as positive control (*%CCND1*/*ABL1 *= 146,85). Only a minimal expression of *CCND1 *(%*CCND1/ABL1 *= 0,37) could be detected for the negative control (Figure 6). In conformity with those findings, western blot analysis of cyclin D1 protein showed a remarkably overexpression (Figure 7).

## Discussion

In general the vast majority of MCL patients show a translocation (11;14)(q13;q32) leading to an *IGH/CCND1 *gene rearrangement. Atypical translocations involving *CCND1 *and immunoglobulin kappa chain (*Igk*) also leading to an overexpression of cyclin D1 have been reported [18]. *IGL *translocations were found in different neoplasms, e.g. t(18;22) leading to *BCL2 *to *IGL *rearrangement in a case of CLL [19] or *c-MYC *to *IGL *rearrangement in Burkitt's lymphoma [20]. Komatsu et al. [4] reported on a MCL case showing an atypical rearrangement t(11;22)(q13;q11). They first speculated this translocation might result in a rearrangement of *CCND1 *to the *IGL *gene locus and confirmed their hypothesis on molecular level in a subsequent publication [21]. The WHO classification points to the possibility of this rare translocation, referring to aforementioned work [22].

In our case we investigated the affected gene on chromosome 22 involved in the t(11;22) and therefore responsible for the cyclin D1 overexpression, possibly leading to the development of an MCL. A FISH analysis with a *BCR/ABL *probe was performed, due to the localization of *BCR *on 22q11 and different reports showing atypical cases of *BCR *translocations to e.g. *FGFR1 *or *PDGFRA *genes [23,24]. As we found a normal signal pattern for *BCR/ABL *and therefore excluded the involvement of *BCR *to the translocation on chromosome 22 we assumed an involvement of *IGL*. FISH analysis as the method of choice for proving *IGL *involvement has already been reported for different chromosome 22 translocations [25]. Thus, our FISH analysis with a *CCND1/IGL *probe could prove the fusion of *CCND1 *to *IGL *concordantly to the findings by Komatsu et al [4,21]. Furthermore, we could show the cyclin D1 overexpression on mRNA and protein level, consolidating the diagnosis of a MCL-like neoplasia.

Analysis on GTG banded chromosome preparations revealed two different aberrant clones. The dominant clone, presumably the mainline, revealed an additional chromosome 3 and the translocation between chromosomes 11 and 22. The second clone showed different additional secondary abnormalities affecting chromosomes X, 1, 8 and 17. As reported before, *CCND1 *translocations normally are considered as the primary cytogenetic event in MCL. Secondary events are seen in up to 88% of MCL cases, often involving trisomy 3, breaks in 1p21 and 17p deletions among others [26], in conformity with our findings in this case. The discovery of double minutes (DM) was quite surprising, as double minutes are very rare in lymphatic neoplasms. DM are acentric extrachromosomal chromatin which may lead to an amplification of oncogenes, like *c-MYC *amplification in colorectal carcinoma [27]. Unfortunately, there was no investigation material left for further analyzing the DM, e.g. by array-CGH to finally clarify the source of this additional genetic material.

## Conclusions

Our patient was first suspected to have a CLL. We could show, that the t(11;22)(q13;q11.2) leads to an overexpression of cyclin D1 due to the rearrangement of *CCND1 *to *IGL*. In addition immunophenotype and morphology of the cells showed a typical MCL configuration. However, some cases of t(11;14) positive CLL [9] and on the other hand t(11;14) negative MCL have been reported [28]. Cyclin D1 is usually overexpressed in MCL, but also overexpression of cyclin D2 or cyclin D3 may induce a MCL [5]. In general, B-cell neoplasms carrying rare translocations involving *CCND1 *and *Ig kappa *or *Ig lambda*, like reported here, are diagnosed as MCL. A distinction between indolent and common cases of MCL has been proposed lately by Fernandez et al. [29]. On the other hand cyclin D1 overexpression in three cases with an *IGK-CCND1 *rearrangement have been diagnosed as small-cell B-non-Hodgkin lymphoma, as they did not show typical features of MCL [18]. In our case we could show some typical features of a MCL like a *CCND1 *translocation, recurrent secondary chromosomal aberrations and cyclin-D1 overexpression. But also some atypical features were found like the existence of double minutes and mutated IgVH status, and therefore we suggest calling this a case of a MCL-like B-cell neoplasia.

Summing up, we described a third case of a t(11;22)(q13;q11.2) leading to a rearrangement of *CCND1 *to *IGL *and showed that this aberration leads to an overexpression of *CCND1 *which is regarded as a key biological feature of MCL. This case underlines the importance of cytogenetic analyses especially in atypical cases of B cell lymphomas.

## Consent

Written informed consent was obtained from the patient for publication of this case report and any accompanying images. A copy of the written consent is available for review by the Editor-in-Chief of this journal.

## Competing interests

The authors declare that they have no competing interests.

## Authors' contributions

CKR performed the cytogenetic and FISH analysis. IP performed the PCR analysis, IG performed the Protein analysis. MH and KAK supervised the experiments and helped in drafting the manuscript. CKR drafted the manuscript and all authors read and approved the final manuscript.

## References

[B1] Morton LM Wang SS Devesa SS Hartge P Weisenburger DD Linet MS Lymphoma incidence patterns by WHO subtype in the United States, 1992-2001 Blood 2006 107 265 276 10.1182/blood-2005-06-2508 16150940 PMC1895348

[B2] Jares P Campo E Advances in the understanding of mantle cell lymphoma Br J Haematol 2008 142 149 165 10.1111/j.1365-2141.2008.07124.x 18410453

[B3] Pileri SA Falini B Mantle cell lymphoma Haematologica 2009 94 1488 1492 10.3324/haematol.2009.013359 19880776 PMC2770958

[B4] Komatsu H Yoshida K Seto M Iida S Aikawa T Ueda R Overexpression of PRAD1 in a mantle zone lymphoma patient with a t(11;22)(q13;q11) translocation Br J Haematol 1993 85 427 429 10.1111/j.1365-2141.1993.tb03194.x 8280621

[B5] Quintanilla-Martinez L Slotta-Huspenina J Koch I Klier M Hsi ED de LL Differential diagnosis of cyclin D2+ mantle cell lymphoma based on fluorescence in situ hybridization and quantitative real-time-PCR Haematologica 2009 94 1595 1598 10.3324/haematol.2009.010173 19608671 PMC2770971

[B6] Salaverria I Espinet B Carrio A Costa D Astier L Slotta-Huspenina J Multiple recurrent chromosomal breakpoints in mantle cell lymphoma revealed by a combination of molecular cytogenetic techniques Genes Chromosomes Cancer 2008 47 1086 1097 10.1002/gcc.20609 18709664

[B7] Mayr C Speicher MR Kofler DM Buhmann R Strehl J Busch R Chromosomal translocations are associated with poor prognosis in chronic lymphocytic leukemia Blood 2006 107 742 751 10.1182/blood-2005-05-2093 16179374

[B8] Decker T Schneller F Kronschnabl M Dechow T Lipford GB Wagner H Immunostimulatory CpG-oligonucleotides induce functional high affinity IL-2 receptors on B-CLL cells: costimulation with IL-2 results in a highly immunogenic phenotype Exp Hematol 2000 28 558 568 10.1016/S0301-472X(00)00144-2 10812246

[B9] Haferlach C Dicker F Schnittger S Kern W Haferlach T Comprehensive genetic characterization of CLL: a study on 506 cases analysed with chromosome banding analysis, interphase FISH, IgV(H) status and immunophenotyping Leukemia 2007 21 2442 2451 10.1038/sj.leu.2404935 17805327

[B10] Wren C Moriarty H Marsden K Tegg E Cytogenetic investigations of chronic lymphocytic leukemia Cancer Genet Cytogenet 2010 198 155 161 10.1016/j.cancergencyto.2009.12.014 20362231

[B11] Bacher U Haferlach T Schnittger S Weiss T Burkhard O Bechtel B Detection of a t(4;14)(p16;q32) in two cases of lymphoma showing both the immunophenotype of chronic lymphocytic leukemia Cancer Genet Cytogenet 2010 200 170 174 10.1016/j.cancergencyto.2010.03.009 20620602

[B12] Karakosta M Tsakiridou A Korantzis I Manola KN Deletion of 5q as a rare abnormality in chronic lymphocytic leukemia Cancer Genet Cytogenet 2010 200 175 179 10.1016/j.cancergencyto.2010.04.002 20620603

[B13] Ho AK Hill S Preobrazhensky SN Miller ME Chen Z Bahler DW Small B-cell neoplasms with typical mantle cell lymphoma immunophenotypes often include chronic lymphocytic leukemias Am J Clin Pathol 2009 131 27 32 10.1309/AJCPPAG4VR4IPGHZ 19095562

[B14] Costa ES Pedreira CE Barrena S Lecrevisse Q Flores J Quijano S Automated pattern-guided principal component analysis vs expert-based immunophenotypic classification of B-cell chronic lymphoproliferative disorders: a step forward in the standardization of clinical immunophenotyping Leukemia 2010 24 1927 1933 10.1038/leu.2010.160 20844562 PMC3035971

[B15] Schaffer LG Slovak ML Campbell LJ editors ISCN 2009: an international system for human cytogenetic nomenclature 2009 Basel: Karger

[B16] Drexler HG MacLeod RA Malignant hematopoietic cell lines: in vitro models for the study of mantle cell lymphoma Leuk Res 2002 26 781 787 10.1016/S0145-2126(02)00026-7 12127550

[B17] Erdfelder F Hertweck M Filipovich A Uhrmacher S Kreuzer K-A High lymphoid enhancer-binding factor-1 expression is associated with disease progression and poor prognosis in chronic lymphocytic leukemia Hematology Reports 2010 24 27 10.4081/hr.2010.e3 PMC3222268 22184516

[B18] Wlodarska I Meeus P Stul M Thienpont L Wouters E Marcelis L Variant t(2;11)(p11;q13) associated with the IgK-CCND1 rearrangement is a recurrent translocation in leukemic small-cell B-non-Hodgkin lymphoma Leukemia 2004 18 1705 1710 10.1038/sj.leu.2403459 15306823

[B19] Adachi M Tsujimoto Y Juxtaposition of human bcl-2 and immunoglobulin lambda light chain gene in chronic lymphocytic leukemia is the result of a reciprocal chromosome translocation between chromosome 18 and 22 Oncogene 1989 4 1073 1075 2506501

[B20] Martin-Subero JI Klapper W Sotnikova A Callet-Bauchu E Harder L Bastard C Chromosomal breakpoints affecting immunoglobulin loci are recurrent in Hodgkin and Reed-Sternberg cells of classical Hodgkin lymphoma Cancer Res 2006 66 10332 10338 10.1158/0008-5472.CAN-06-1992 17079453

[B21] Komatsu H Iida S Yamamoto K Mikuni C Nitta M Takahashi T A variant chromosome translocation at 11q13 identifying PRAD1/cyclin D1 as the BCL-1 gene Blood 1994 84 1226 1231 8049438

[B22] Swerdlow SH Campo E Harris NL Jaffe ES Pileri SA Stein H WHO classification of tumours of haematopoietic and lymphoid tissues 2008 Lyon: IARC

[B23] Demiroglu A Steer EJ Heath C Taylor K Bentley M Allen SL The t(8;22) in chronic myeloid leukemia fuses BCR to FGFR1: transforming activity and specific inhibition of FGFR1 fusion proteins Blood 2001 98 3778 3783 10.1182/blood.V98.13.3778 11739186

[B24] Baxter EJ Hochhaus A Bolufer P Reiter A Fernandez JM Senent L The t(4;22)(q12;q11) in atypical chronic myeloid leukaemia fuses BCR to PDGFRA Hum Mol Genet 2002 11 1391 1397 10.1093/hmg/11.12.1391 12023981

[B25] Martin-Subero JI Harder L Gesk S Schlegelberger B Grote W Martinez-Climent JA Interphase FISH assays for the detection of translocations with breakpoints in immunoglobulin light chain loci Int J Cancer 2002 98 470 474 10.1002/ijc.10169 11920602

[B26] Espinet B Salaverria I Bea S Ruiz-Xiville N Balague O Salido M Incidence and prognostic impact of secondary cytogenetic aberrations in a series of 145 patients with mantle cell lymphoma Genes Chromosomes Cancer 2010 49 439 451 20143418 10.1002/gcc.20754

[B27] Shimizu N Extrachromosomal double minutes and chromosomal homogeneously staining regions as probes for chromosome research Cytogenet Genome Res 2009 124 312 326 10.1159/000218135 19556783

[B28] Stefancikova L Moulis M Fabian P Falkova I Vasova I Kren L Complex analysis of cyclin D1 expression in mantle cell lymphoma: two cyclin D1-negative cases detected J Clin Pathol 2009 62 948 950 10.1136/jcp.2008.063701 19783727

[B29] Fernandez V Salamero O Espinet B Sole F Royo C Navarro A Genomic and gene expression profiling defines indolent forms of mantle cell lymphoma Cancer Res 2010 70 1408 1418 10.1158/0008-5472.CAN-09-3419 20124476

